# Class III Peroxidases (POD) in Pepper (*Capsicum annuum* L.): Genome-Wide Identification and Regulation during Nitric Oxide (NO)-Influenced Fruit Ripening

**DOI:** 10.3390/antiox12051013

**Published:** 2023-04-27

**Authors:** Salvador González-Gordo, María A. Muñoz-Vargas, José M. Palma, Francisco J. Corpas

**Affiliations:** Group of Antioxidants, Free Radicals and Nitric Oxide in Biotechnology, Food and Agriculture, Department of Stress, Development and Signaling in Plants, Estación Experimental del Zaidín, Spanish National Research Council (CSIC), C/Profesor Albareda 1, 18008 Granada, Spain; salvador.gonzalez@eez.csic.es (S.G.-G.); mangeles.munoz@eez.csic.es (M.A.M.-V.); josemanuel.palma@eez.csic.es (J.M.P.)

**Keywords:** fruit ripening, nitric oxide, nitration, peroxidase, pepper

## Abstract

The class III peroxidases (PODs) catalyze the oxidation of several substrates coupled to the reduction of H_2_O_2_ to water, and play important roles in diverse plant processes. The POD family members have been well-studied in several plant species, but little information is available on sweet pepper fruit physiology. Based on the existing pepper genome, a total of 75 *CaPOD* genes have been identified, but only 10 genes were found in the fruit transcriptome (RNA-Seq). The time-course expression analysis of these genes showed that two were upregulated during fruit ripening, seven were downregulated, and one gene was unaffected. Furthermore, nitric oxide (NO) treatment triggered the upregulation of two *CaPOD* genes whereas the others were unaffected. Non-denaturing PAGE and in-gel activity staining allowed identifying four CaPOD isozymes (CaPOD I-CaPOD IV) which were differentially modulated during ripening and by NO. In vitro analyses of green fruit samples with peroxynitrite, NO donors, and reducing agents triggered about 100% inhibition of CaPOD IV. These data support the modulation of POD at gene and activity levels, which is in agreement with the nitro-oxidative metabolism of pepper fruit during ripening, and suggest that POD IV is a target for nitration and reducing events that lead to its inhibition.

## 1. Introduction

Peroxidases (PODs) constitute a heterogeneous group of enzymes that catalyze the oxidation of a large variety of substrates using hydrogen peroxide (H_2_O_2_) as an oxidizing agent. Being widely distributed in all living organisms, PODs can be classified according to their structural and catalytic properties into different families [1]. Among them, class III peroxidases (EC 1.11.1.7) are enzymes exclusive to the plant kingdom. In higher plants, a huge diversity of POD isozymes encoded by a multigene family has been reported [2,3,4]. Although the abbreviation used in the literature varies as POD or PRX, in this study we have used POD to avoid possible confusion with another group of peroxidases, namely peroxiredoxins (Prx; EC 1.11.1.15), which will not be included in present analyses.

The number of class III POD-encoding genes varies according to the species, ranging from 47 in grape (*Vitis vinifera*) [5], 73 in *Arabidopsis thaliana* [6], 82 in sugarcane (*Saccharum spontaneum*) [7], 102 in potato (*Solanum tuberosum*) [8], and 124 *POD* genes in soybean (*Glycine max* L.) [9] to 138 *POD* genes in rice (*Oryza sativa*) [10], or even, more than 200 in tobacco (*Nicotiana tabacum*) [11]. Considering their participation in lignification and suberization processes as well as their induction in response to pathogen attacks, POD functions were mainly associated with plant defense mechanisms. However, different studies have suggested that POD enzymes are involved in a wide range of physiological processes [12], including seed germination [13], pollen development [14], pollination [15], phytohormone metabolism [16] and fruit ripening [17,18,19], as well as in the mechanism of abiotic stresses [9,20,21]. Furthermore, POD activity is considered an enzymatic indicator of quality deterioration in fruits since it is associated with bad taste [22,23,24].

Pepper (*Capsicum annuum* L.) is the most widespread and cultivated species of the Capsicum genus, belonging to the Solanaceae family. Besides its agronomic importance, pepper fruit is gaining great relevance as a nutraceutical product due to its high content of antioxidants, mainly vitamins A and C, and other compounds [25,26,27]. Additionally, some pepper varieties contain a group of specific alkaloids, known as capsaicinoids, with capsaicin being the most abundant one. These molecules, which are responsible for the pungency of hot peppers, have beneficial effects on human health [28,29,30]. One of the most relevant aspects of fruit physiology is the ripening process. Once the sweet pepper fruit has reached its final developmental size, which could take around two weeks, it begins its ripening phase from green to fully red ripe within the next 3 to 7 days. Commercial ripening starts when a very small part of the fruit shifts from green to a brownish/reddish color that is progressing to entirely red at the final stage. A characteristic of green peppers, as climacteric fruits, is that they cannot ripen off the plant, unless ripening has started while they are on the plant. In any case, sweet pepper fruits are edible at any stage of ripening, depending on the culinary practices in use. The metabolic alterations that take place during fruit development, combined with postharvest management, can determine the quality and nutritional properties of the fruit. Previous studies have shown that numerous changes in nitro-oxidative metabolism occur during pepper fruit ripening, where molecules such as reactive oxygen and nitrogen species (ROS and RNS, respectively) play key roles [31,32]. In this context, nitric oxide (NO) seems to be a master regulator in this physiological process at different levels. On the one hand, it has been observed that the external application of NO gas caused a delay in ripening associated with an increase in the antioxidant capacity of the fruit [33,34,35]. Furthermore, NO can endogenously modulate the activity of certain enzymes through post-translational modifications (PTMs) such as nitration and *S*-nitrosation. In previous studies, we have analyzed some components involved in the metabolism of ROS and RNS including catalase [36], superoxide dismutase [37], ascorbate peroxidase [38], superoxide (O_2_^•−^)-generation by an NADPH oxidase [39], lipoxygenase [40], nitrosoglutathione reductase [41], as well as different NADPH-generating enzymes [42,43,44] and small heat shock proteins [45]. However, to our knowledge, there has not been any research study focused on the family of type III peroxidases (PODs) in sweet pepper fruits.

Therefore, this study aims to identify and characterize the genes that code for PODs in pepper fruits, and to analyze their expression during ripening and as result of the exogenous application of NO gas. The obtained data indicate the presence of 10 *CaPOD* genes in fruit that are differentially regulated. Likewise, the zymogram of POD activity shows the presence of four isozymes (CaPOD I–CaPOD IV), and in vitro assays demonstrated that the CaPOD IV was significantly regulated by RNS (nitration and *S*-nitrosation) and reducing agents such as glutathione and L-cysteine.

## 2. Materials and Methods

### 2.1. Identification of POD Genes in Pepper, Chromosomal Location and Synteny Analysis

To identify the different peroxidase Class III-encoding genes in pepper (*C. annuum* L.), we carried out two different approaches. On the one hand, pepper proteome was downloaded from the NCBI database (Assembly UCD10Xv1.1; BioProject PRJNA814299; https://www.ncbi.nlm.nih.gov/bioproject/814299, accessed on 10 February 2023). The amino acid sequences from the PODs described in the model plant Arabidopsis were downloaded from the UnirProtKB database (https://www.uniprot.org/help/uniprotkb, accessed on 10 February 2023). These sequences were used as queries to search for PODs in the complete pepper proteome using the BLASTP v2.2.30 tool. On the other hand, we used the HMMER v3.3.2 software [46] to search for proteins that conserved the peroxidase domain in their sequence (PF00141), which was previously downloaded from the Pfam database [47]. Finally, redundant proteins, proteins identified as ascorbate peroxidases (APXs), and proteins whose length was out of the range of 300–400 amino acids were manually checked and discarded. 

Location coordinates of the identified *CaPOD* genes in the pepper genome were obtained from the NCBI database. The co-localization analysis of the different *CaPODs* among the pepper chromosomes was performed using the toolkit MCScanX v1.0.0 [48]. This information was plotted in a genomic map elaborated with the software TBtools v1.108 [49] using the ‘Advanced Circos’ function. 

### 2.2. Phylogenetic and Conserved Motif Analyses of POD Protein Sequences

The identified POD protein sequences in pepper and Arabidopsis were used to construct a phylogenetic tree. The alignment of PODs was performed using the CLUSTALW method [50]. Then, the aligned sequences were subjected to MEGA11 v0.13 [51] to perform an unrooted maximum likelihood phylogenetic tree with default parameters. Finally, the resulting phylogenetic tree was modified using the online tool Evolview v3 [52]. Conserved motifs of CaPODs were sought using the MEME tool [53] and visualized using TBtools software v1.108 [49]. The protein localization based on their amino acid sequences was predicted using WoLF PSORT (https://wolfpsort.hgc.jp/, accessed on 10 February 2023) [54].

### 2.3. Analysis of the CaPODs Cis-Regulatory Elements

The promoter sequences of the *CaPODs* were obtained from the NCBI Nucleotide database (https://www.ncbi.nlm.nih.gov/nucleotide/; accessed on 10 February 2023) considering 1500 bp upstream from the transcription starting point of each gene. These sequences were searched for possible cis-acting regulatory elements using the PantCARE tool (https://bioinformatics.psb.ugent.be/webtools/plantcare/html/, accessed on 10 February 2023) [55]. These results were manually processed and visualized using the ‘Basic Biosequence View’ function of the TBtools v1.108 software [49]. 

### 2.4. Plant Material and Exogenous Nitric Oxide (NO) Gas Treatment

Sweet pepper (*C. annuum* L. cultivar Melchor) fruits belonging to the California type were assayed. For several years, fruits were harvested between January and February from plants grown in plastic-covered greenhouses (Zeraim Iberica/Syngenta Seeds, Ltd., Roquetas de Mar/El Ejido, Almería, Spain). Pepper plants were cultivated according to the usual crop program designed by the company: planting and seed germination start in July–August, flowering starts in late September, and pollination and fruit setting take place from middle October to late November. Fruits were harvested from late January to early February. The external average daily temperature, including values from fruit setting (about October 15th) until fruit harvesting (15 January–15 February), was 14.9 ± 0.3 °C. Data were obtained from the Meteorological Station of La Mojonera, Almería, Spain (latitude 36°47′14″ N, longitude 02°42′15″ W), located near the experimental greenhouse where fruits were grown and harvested. 

**Figure 1:**
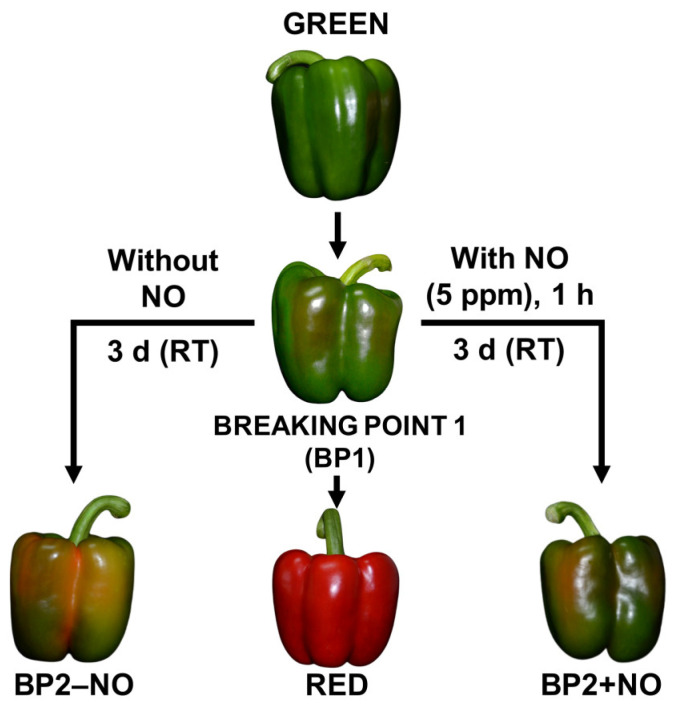
Representative model of the experimental design used in this study with the phenotype of sweet pepper (*C. annuum* L.) fruits at different stages and treatments: immature green, breaking point 1 (BP1), breaking point 2 without nitric oxide (NO) treatment (BP2 − NO), breaking point 2 with NO treatment (BP2 + NO), and ripe red. Pepper fruits were subjected to a NO-enriched atmosphere (5 ppm) in a methacrylate box for one hour and then were stored at room temperature (RT) for 3 days. Reproduced from [35] with permission from Oxford University Press Journal and Copyright Clearance Center (2019).

Fruits from the same plant and without any external apparent injury were selected at three developmental stages: green immature (G), breaking point (BP1), and red ripe (R). Once harvested, the fruits were placed into black plastic bags and taken to the laboratory at room temperature, washed with distilled water, and maintained for 24 h at a low temperature (about 7 °C ± 1 °C). For the analysis of the exogenous NO gas treatment, we set two additional groups: fruits treated with 5 ppm NO for 1 h (BP2 + NO), and fruits that were not treated with NO (BP2 − NO). After 3 days at room temperature, all fruits were chopped into small cubes (5 mm/edge), frozen under liquid nitrogen, and stored at −80 °C until use. Figure 1 shows the experimental design followed in this study with the representative phenotypes of sweet pepper fruits at different ripening stages and subjected to NO treatment [35].

### 2.5. Library Preparation and RNA-Sequencing

All procedures were performed as previously described in [35] with minor modifications. Briefly, libraries were prepared using an Illumina protocol and were sequenced on an Illumina NextSeq550 platform (Illumina, Inc., San Diego, CA, USA) using 2 × 75 bp paired-end reads. These reads were preprocessed to remove low-quality sequences. Useful reads were mapped against the set of transcripts available for *C. annuum* species in the NCBI database (assembly UCD10Xv1.1; https://www.ncbi.nlm.nih.gov/assembly/GCF_002878395.1, accessed on 10 February 2023) using Bowtie2 v2.5.1 [56]. Transcript counts were obtained using Samtools v1.3.1 [57].

Differential expression analyses were done using DEgenes-Hunter v0.98 [58]. This R pipeline examined the relative change in expression between the different samples using different algorithms (EdgeR, DESeq2, Limma, and NOISeq) which applied their own normalizations and statistical tests to validate the whole experiment. On the other hand, an analysis of the time course of *CaPODs*’ gene expression was performed, considering as references the expression levels found in green fruits (G). Raw data are accessible at the Sequence Read Archive (SRA) repository under the accession PRJNA668052. This reference pepper fruit transcriptome, and differentially expressed (DE) genes among the analyzed ripening stages and the NO treatment, involved the analysis of 24 biological replicates corresponding to 5 replicates for each group, except for green fruits, which included 4 replicates.

### 2.6. Preparation of Fruit Crude Extracts for Non-Denaturing PAGE and POD Activy Assay

Frozen samples of sweet pepper fruits at the different ripening stages and after NO treatment were powdered under liquid nitrogen using an IKA^®^ A11Basic analytical mill (IKA^®^, Staufen, Germany), and then extracted in 100 mM Tris-HCl buffer, pH 8.0, containing 0.1% (*v*/*v*) Triton X-100, 1 mM ethylenediaminetetraacetic acid (EDTA), 10% (*v*/*v*) glycerol in a final 1:1 (*w*/*v*) plant material:buffer ratio. The obtained homogenates were centrifuged at 15,000× *g* for 30 min at 4 °C and the supernatants were used for enzymatic POD assays. The protein concentration of the samples was determined with the Bio-Rad Protein Assay (Hercules, CA, USA) using bovine serum albumin as a standard.

Peroxidase (POD) isozymes were separated by non-denaturing PAGE on 8% polyacrylamide gels and the activity was detected as previously described by [59]. Gels were incubated for 20 min in sodium acetate buffer 0.1 M, pH 5.5, containing 3,3-diaminobenzidine 1 mM and H_2_O_2_ (0.03%, *v*/*v*). Brown bands appeared over a colorless background at the end of the reaction. The specificity of the POD isozymes was corroborated because, in the absence of H_2_O_2_, no bands were detected.

### 2.7. In Vitro Treatment Pepper Green Fruit Samples with Different Chemical Agents

For the in vitro assays, samples from green pepper fruits were pre-incubated with different potential modulators including: 3-morpholinosydnonimine (SIN-1), a peroxynitrite (ONOO^−^) donor and nitrating compound; *S*-nitrosoglutathione (GSNO) and *S*-nitrosocysteine (CysNO) as NO donors; L-cysteine (L-Cys) and reduced glutathione (GSH), as reducing compounds; sodium hydrosulfide (NaHS), as H_2_S donor; and the oxidizing compound hydrogen peroxide (H_2_O_2_). In all cases, the solutions were freshly prepared before use at a concentration of 5 mM, and the treatments were done at 25 °C for 1 h in the dark, except the treatment with SIN-1 which was at 37 °C for 1 h.

## 3. Results and Discussion

### 3.1. Pepper Genome Contains 75 POD Genes but Only 10 Genes Are Expressed in Pepper Fruits

**Figure 2:**
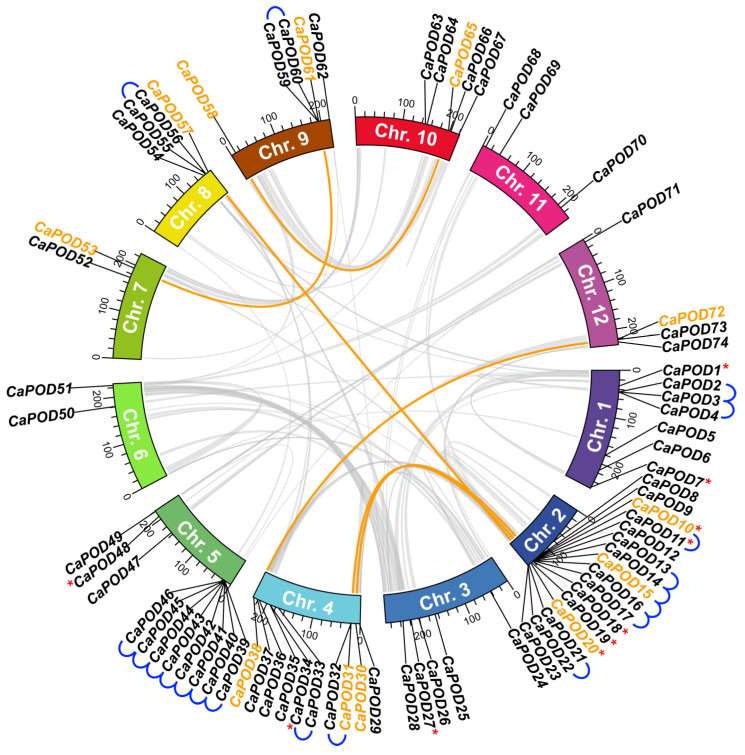
Synteny analysis of *CaPOD* genes. Pepper chromosomes (Chr. 1–12) are represented in different colors. Grey curved lines within the scheme refer to the co-localization of genetic loci in the pepper genome. Collinear relationships between *CaPOD* genes are drawn in orange. Tandem duplication for *CaPOD* genes is indicated with blue lines. Red asterisks indicate those *CaPOD* genes identified in the transcriptome of sweet pepper fruit.

In this study, a total of 75 *POD* genes were identified and characterized in the pepper genome and were designated *CaPOD1*–*CaPOD75* based on their chromosomal locations, and, among them, 10 were found to be exclusively expressed in fruits (Table 1). This number of genes is similar to that found in Arabidopsis, which has 73 [6] but less than that from other Solanaceae species such as potato with 102 [8] or tobacco with 200 POD genes [11]. These 75 *CaPOD* genes were distributed across 12 pepper chromosomes (Figure 2). The encoded CaPOD proteins have a mean molecular mass of 35.6 kDa which is within the range of class III PODs whose subunit size usually ranges from 30–45 kDa [60,61,62]. On the other hand, the subcellular localization of the CaPODs is very diverse with 35 being assigned to plastids, 22 as extracellular, 11 with dual localization (extracellular/cytosol, plastid/vacuole, plastid/Golgi, and plastid/cytosol), 3 in the vacuole, and one in the cytosol (CaPOD28), nucleus (CaPOD37), cytoskeleton (CaPOD65) and peroxisome (CaPOD33). These data are in agreement with previous reports which describe this wide distribution in other plant species [63,64,65,66,67,68].

As previously mentioned, PODs perform various functions which usually are associated with their cellular location. Those that are located in the cytosol and plastids are involved in auxin metabolism, senescence, several biosynthetic pathways, and stress-related processes [18,60]. 

In the case of hot peppers, it has been described that PODs intervene in the oxidation of capsaicin [69]. Extracellular/apoplastic PODs are very closely related to the apoplastic H_2_O_2_ since they participate in cell expansion, development, as well as in defense mechanisms through the formation of polymer cross-linking, lignification, and cell expansion [70]. On the other hand, the vacuole is considered a sink of H_2_O_2_ that enters through aquaporins. This cellular compartment also contains a significant number of PODs that, through phenolic substrates, can oxidize this H_2_O_2_ with the concomitant generation of the corresponding phenoxyl radical that would be reduced by ascorbate [71].

The analysis of the chromosomal localization of these 75 *CaPOD* genes is illustrated in Figure 2, where the 10 genes identified in the pepper fruit transcriptome are marked with red asterisks. These genes showed an irregular distribution pattern since the number of genes on each chromosome was dissimilar. The highest number of genes (16) was found in Chr. 2, followed by Chr. 5 with 11 genes, Chr. 4 with 10, Chr 1 that has 7, Chrs. 9 and 10 have 5, 4 genes were present in Chrs. 8 and 12, while Chr. 6 and Chr. 7 harbor 2 genes. Only *CaPOD75* could not be assigned to any chromosome. A total of 12 tandem genes were detected in pepper Chr. 2 and contain the highest number, with 7 tandem genes (CaPOD39 to CaPOD46) that encode for plastidial PODs. Tandem gene duplication has been considered one of the major reported gene duplication mechanisms in other plant species. Thus, 15 POD tandem genes in grapevine (*Vitis vinifera* L.) [5], 16 *POD* genes in cassava [62], 24 *POD* genes in maize [72], and 37 *POD* genes in *Populus trichocarpa* [73] have been reported. Figure 2 also shows the co-localization of genetic loci (curved lines) which allows sharing of a genetic factor at a particular locus between two or more characters. All these mechanisms could be considered as strategies of adaption and diversification to diverse external adverse conditions.

**Figure 3:**
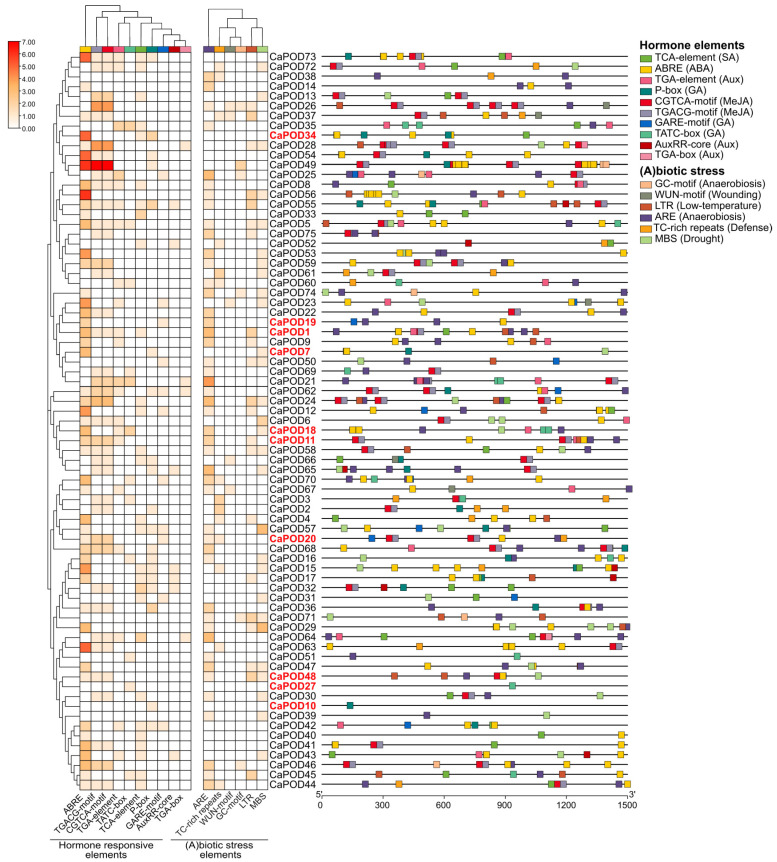
Heatmap of cis-regulatory elements corresponding to the 1500 bp upstream region from the transcription start point of *CaPOD* genes. The distribution of these elements in the promoter sequence is also shown. The cis-regulatory elements were grouped according to their functional implications as hormone-responsive elements and abiotic/biotic stress-responsive elements. *CaPOD* genes identified in the sweet pepper fruit transcriptome are highlighted in red. Motifs were identified from the PlantCARE database.

As part of the characterization of the *CaPOD* genes, the presence of cis-regulatory elements in 1500 bp upstream regions from the transcription starting point of the 75 *CaPOD* genes was evaluated. Figure 3 shows the heatmap analysis of the identified 16 cis-regulatory elements which were clustered into two groups corresponding to hormone-responsive and (a)biotic stress elements. The cis-regulatory element which exerted the most remarkable effect was that of the group of hormone-responsive elements. Thus, ABRE (ACGT-containing abscisic acid response element), which is involved in abscisic acid (ABA) responsiveness, affected *CaPOD15*, *CaPOD49*, *CaPOD56*, *CaPOD63*, and *CPOD73*. On the other hand, the promoter sequences of *CaPOD24*, *CaPOD26*, and *CaPOD49*.

It has been reported that ABA content is modulated during pepper ripening, and total carotenoid content, which increased during ripening, was ABA-dependent [74]. Thus, among the ten POD genes that are expressed in fruit, CaPOD34 was the one with the highest ABRE expression, followed by CaPOD1 and CaPOD7.

**Figure 4:**
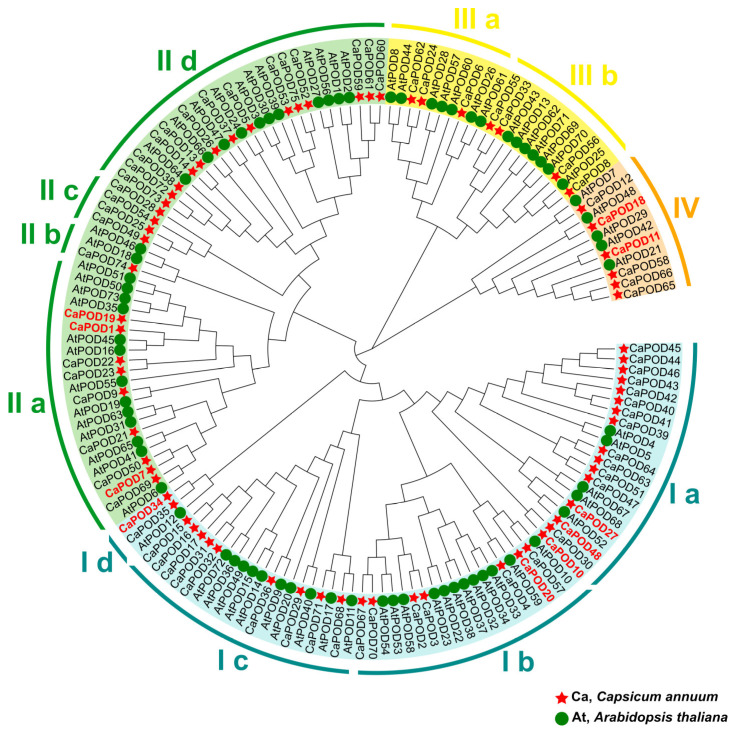
Phylogenetic relationships between pepper and Arabidopsis *POD* genes. Clusters (I–IV) are displayed in different colors. Clusters were divided into different subgroups (a–d) according to their evolutionary distance. Species abbreviations: At (*Arabidopsis thaliana*), Ca (*Capsicum annuum*). Those CaPODs identified in the transcriptome of sweet pepper fruit are highlighted in red.

To examine the evolutionary relationships among genes, we used the identified 75 *CaPOD* genes and 73 *AtPOD* genes from the model plant *Arabidopsis thaliana* to construct a maximum likelihood approach tree by using MEGA11. The phylogenetic relationships reveal that *CaPOD* genes can be further categorized into 4 clusters designated from CI to CIV (Figure 4). It should be noted that the *CaPOD* genes expressed in fruits (written in red in Figure 3) are differently distributed in these groups. Thus, the group I includes CaPOD10, CaPOD20, CaPOD27, CaPOD34, and CaPOD48; group II includes CaPOD1, CaPOD7, and CaPOD19; and group IV includes CaPOD11 and CaPOD18. 

**Figure 5:**
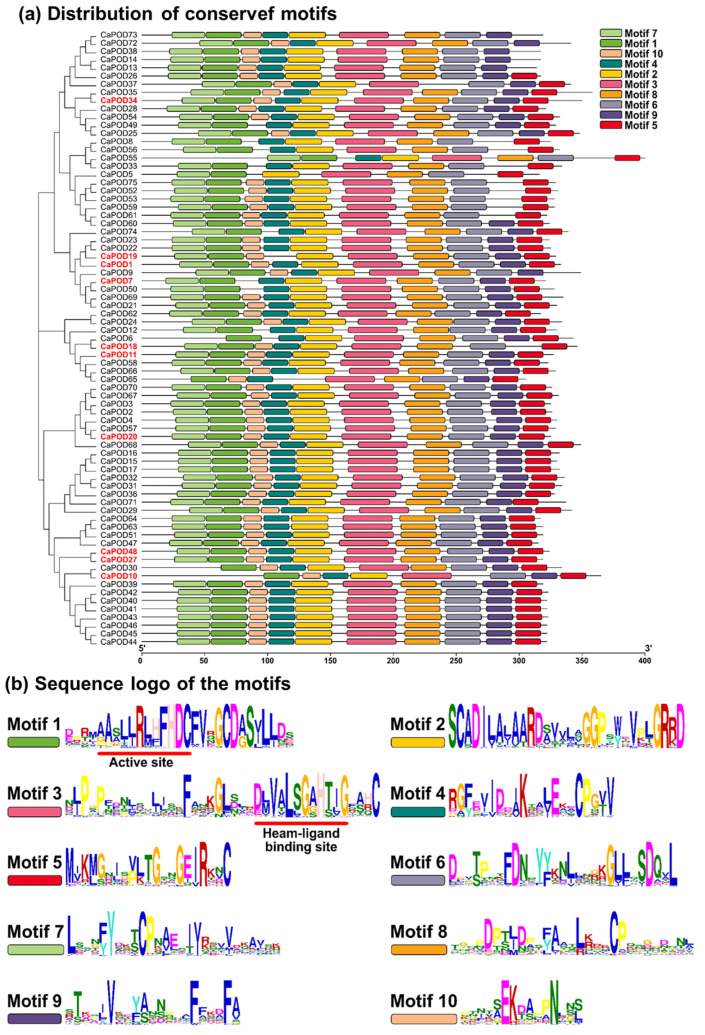
Identification and position of consensus amino acid motifs for pepper CaPODs. (**a**) Distribution of conserved motifs. The distribution of conserved motifs numbered 1–10 of the 75 pepper PODs is represented by boxes of different colors. (**b**) Amino acids sequence of the motifs. Ten amino acid motifs with various were identified and are represented with different sizes. The height of each amino acid symbol is proportional to the degree of conservation in the consensus sequences depicted in the ten motifs. Sequence logos of conserved motifs were created by MEME.

At the amino acid sequence level, the identified 75 CaPODs class III have an average of 330 residues (see Table 1). The alignment of these CaPODs allows to establish a total of 10 conserved motifs (Figure 5a), whose amino acid sequences are shown in Figure 5b. The most remarkable issue from the sequences is that motif 1 holds the active site, whereas motif 3 includes the heam-ligand binding site for the Fe(III) protoporphyrin IX. Furthermore, the class III peroxidase contains two calcium atoms and has a similar 3-D structure with 4 disulfide bonds, based on the conserved Cys residue pairs, and a salt bridge motif containing invariant Asp and Arg residues [68,75].

### 3.2. The Expression of the CaPOD Genes Is Mainly Downregulated during Fruit Ripening. Exogenous NO Gas Only Exerts a Positive Modulation of CaPOD 13, 37, and 39

**Figure 6:**
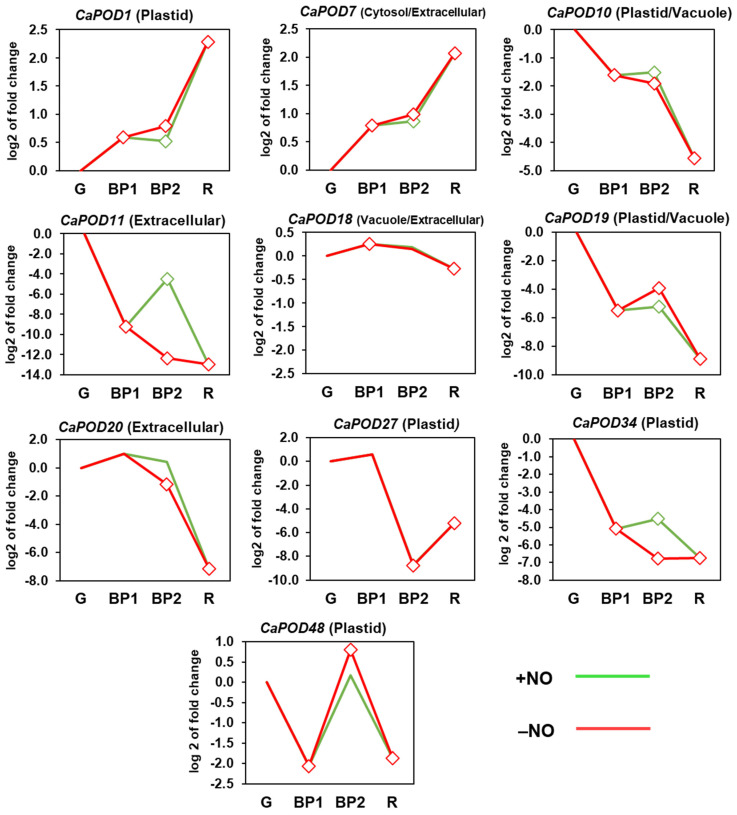
Time-course expression analysis of *CaPOD* genes (RNA-Seq) from pepper fruits. Differences in temporal expression patterns between different ripening stages of sweet pepper fruit and the effect of NO treatment on genes encoding different POD isoforms are shown. Samples corresponding to immature green (G), breaking point 1 (BP1), breaking point 2 with and without NO treatment (BP2 + NO and BP2 − NO, respectively), and red (R) were used. Diamonds indicate statistically significant changes in expression levels (*p* < 0.05) in comparison to immature green fruits (G). Green line: BP2 fruits treated with NO. Red line: untreated fruits.

Figure 6 shows the time-course expression analysis of *CaPOD* genes during ripening. Thus, *CaPOD1* and *CaPOD7* were upregulated, whereas *CaPOD10*, *CaPOD11*, *CaPOD19*, *CaPOD20*, *CaPOD27*, *CaPOD34*, and *CaPOD48* were downregulated, and only *CaPOD18* was unaffected. On the other hand, the effect of NO was positive in *CaPOD11* and *CaPOD34*, while the other *CaPOD* genes were not significantly affected by this RNS. These data indicate that most *CaPODs* may play a role in the fruit ripening process, whereas *CaPOD11* and *CaPOD34* could be involved in the effect provoked by treatment with NO.

In the Chinese pear (*Pyrus bretschneideri*) a total of 94 *PbPOD* genes have been identified, from which 41 were expressed in fruits, and among them, a group of 5 *PbPOD* genes, particularly *PbPOD2*, *PbPOD22*, *PbPOD34*, *PbPOD64*, and *PbPOD75* were associated to a change in the content of lignin during the development of the fruits [19,76]. On the other hand, the information on the exogenous application of NO in fruit ripening indicates that this gas molecule affects multiple enzymatic systems which are involved in H_2_O_2_ decomposition such as catalase or ascorbate peroxidases. However, to the best of our knowledge, the effect of exogenous NO on the expression of *CaPOD* genes during fruit ripening has not been studied thus far.

### 3.3. Pepper Fruits Have Four CaPOD Isozymes (CaPOD I–IV) and POD IV Is Significantly Modulated by NO-Derived Molecules and Reducing Agents

**Figure 7:**
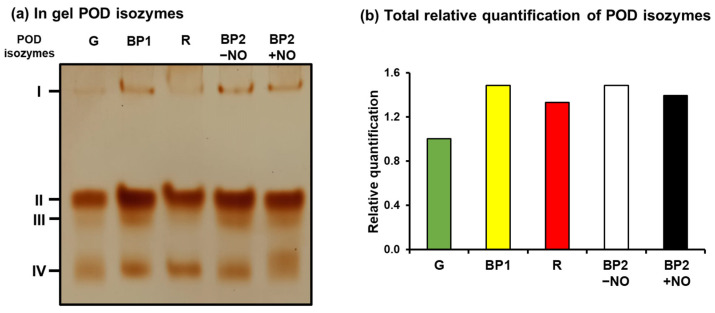
Isozyme peroxidase (POD) activity of sweet pepper fruits at different ripening stages: immature green (G), breaking point 1 (BP1), breaking point 2 with and without NO treatment (BP2 + NO and BP2 − NO, respectively), and red ripe (R). (**a**) In-gel isozyme profile of POD activity). (**b**) Total relative quantification of POD isoenzyme activity. Protein samples (28 µg per lane) were separated by non-denaturing polyacrylamide gel electrophoresis (PAGE; 8% acrylamide), and the activity was detected by the 3,3-diaminobenzidine method. POD isozymes were labeled I–IV, according to their increasing electrophoretic mobility.

To get deeper insights into the function of the PODs during pepper fruit ripening and the effect of NO, an analysis was made at the enzymatic activity level. Figure 7a shows the in-gel staining POD isozymes analysis in pepper fruits at different ripening stages: immature green (G), breaking point (BP1), BP2 with and without NO (BP2 + NO and BP2 − NO, respectively), and ripe red (R), as previously characterized (Figure 1). Four POD isozymes were identified in the assays, designated CaPOD I to CaPOD IV, according to their increasing electrophoretic mobility in the gel, and it was observed that they were slightly modulated during ripening and by NO. Figure 7b depicts the relative activity quantification considering all the POD isozymes present at each ripening stage. Thus, a slight increase in the total activity in red pepper compared to green was observed. On the other hand, in the intermediate stage (BP2), the effect of NO in total activity seems to be not significant, although POD IV seems to be positively modulated.

The number of POD isozymes is highly variable depending on both the plant species as well as the organ analyzed. For example, in the halophyte *Cakile maritima*, four POD isozymes have been found in leaves and 5 PODs in roots, and they were differently modulated under salinity and potassium deficiency [77]. During the development and ripening of tomato fruits, it has been described that the number of POD isozymes is different in the mesocarp and in the skin. Thus, in the skin, the number of POD isozymes increased from one to 4 PODs, whereas in the mesocarp, the increase was from 1 to 2 POD isozymes. In both cases, the total POD activity was enhanced, this being associated with the regulation of fruit growth by cross-linking cell wall polymers within the tomato skin, thus mechanically thickening the walls and terminating growth [64,72,78,79]. In the case of pepper fruit *C. annuum* cv. Padrón, whose capsaicinoid content increases during ripening, this phenomenon was associated with a decrease in total POD activity, where the acidic POD isozymes increased while the basic POD isozymes decreased [80].

**Figure 8:**
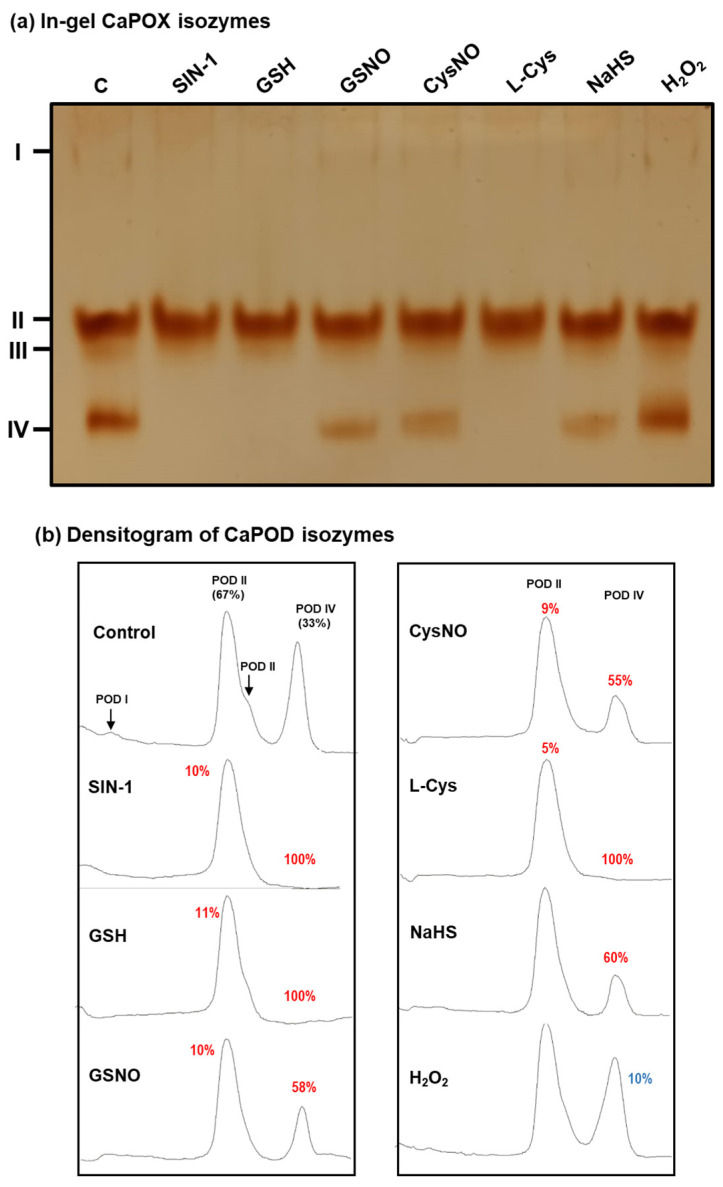
Effect of nitration, *S*-nitrosation, and reducing agents on the green pepper fruit peroxidase (POD) isozymes analyzed in non-denaturing gels. (**a**) In-gel isozyme profile of POD activity in 8% acrylamide gels. (**b**) Densitometric analysis of POD isozymes and their relative quantification (%) was made by the ImageJ program. SIN-1 is a peroxynitrite donor and a nitrating compound. GSNO (S-nitrosoglutathione) and CysNO (nitrosocysteine) are NO donors and nitrosating agents. L-Cys: cysteine. GSH: reduced glutathione. NaHS: sodium hydrosulfide as H_2_S donor. H_2_O_2_: hydrogen peroxide. All treatments were done by pre-incubating the green pepper samples (28 µg protein per lane) with these compounds (5 mM) at 25 °C for 1 h, except with SIN-1, which was pre-incubated at 37 °C for 1 h. The number assigned to each peak indicates the percentage of either isozyme activity inhibition (red) or activation (blue) in relation to the control samples (green fruit crude extracts) after the quantification made with the help of the ImageJ program.

To gain deeper insights into how each specific POD isozyme might be influenced by the NO treatment, in vitro analyses with different potential modulators on the activity of the POD isozymes identified in green pepper fruits were carried out by non-denaturing PAGE (Figure 8a). In this case, we focused on CaPOD II and IV because they were the most prominent isozymes. The relative quantification of each POD isozyme showed that the CaPOD II was slightly inhibited (around 10%) by the nitrating agent SIN-1, an ONOO^−^ donor, by reduced glutathione (GSH), by the NO donors nitrosoglutathione (GSNO) and nitrosocysteine (CysNO), but it was unaffected by NaHS or H_2_O_2_. However, the effect of these potential modulators was most evident in the case of CaPOD IV, since a full inhibition by SIN-1, GSH, and Cys was observed, about 58% and 55% inhibition by the NO donors GSNO and L-CysNO, respectively, and 60% by NaHS (Figure 8b). On the other hand, H_2_O_2_ increased POD IV activity by 10%. To our knowledge, this is the first report showing clear experimental evidence of the interaction of NO-derived molecules on specific POD isozymes in plants.

The fact that POD IV is inhibited 100% by GSH and Cys, and only around 50% by the NO donors, GSNO and CysNO, which, when are chemically dissociated, release NO, and GSH and Cys, respectively, suggests that NO could trigger a process of *S*-nitrosation of POD IV, and this might exert a protective effect against the inhibitory effect of GSH and Cys. Recently, a similar response was observed in the H_2_S-generating enzymes L-Cys desulfhydrase activity of pepper fruits [81]. 

This inhibitory effect of Cys has been described in other enzymes like α-chymotrypsin [82]. There are also some examples showing the effect of L-Cys on peroxidase activity. Thus, 0.25% L-Cys reduced the activities of POD and polyphenol oxidase (PPO) enzymes in litchi fruit thus delaying pericarp browning after postharvest [83]. A similar inhibitory effect of L-Cys has been observed on polyphenol oxidase activity avoiding the browning of pear [84] and peach [85] by lowering pH and chelating copper ions. Likewise, the application of L-Cys at 0.5% concentration inhibits polyphenol oxidase activities and alleviates internal browning in plum (*Prunus domestica* L.) fruit during its storage at low temperatures (1 °C) [86].

At present, it is well recognized that the exogenous application of NO exerts its beneficial effects on fruit quality, particularly during postharvest storage, influencing in many cases the ROS metabolism [87]. The negative effect of NO on the POD activity has been observed in different fruits treated with different NO donors. For example, the browning of fresh-cut potatoes is related among other enzymes to the POD activity. However, the treatment with the NO donor sodium nitroprusside triggers a lower POD activity in fresh-cut potatoes preventing browning events [88]. In kiwifruit (*Actinidia chinensis* Planch. cv. Xuxiang) treated with a solution of 1 µM NO the POD activity was lower than that in control fruit throughout storage, and this was associated with a lower H_2_O_2_ level. In fact, the NO treatment also triggered higher activities of superoxide dismutase (SOD) and catalase, as well as a high content of vitamins C and E, thus contributing to lower H_2_O_2_ and malondialdehyde contents. Consequently, all these effects allowed the preservation of the quality of the kiwifruit [89]. The inhibitory effect of L-Cys has been observed in different enzymes. In papaya, guaiacol peroxidase was inhibited by Cys, and modelling assays with the enzyme applying docking analyses with various substrates and inhibitors, it was found that guaiacol and cysteine were the best substrate and inhibitor, respectively [90].

On the other hand, the S-nitrosation is claimed to be a mechanism of protection of thiol groups of some key Cys residues that could be involved in the active center of the target proteins [91]. This supports the idea of the relevance of the redox state of the protein’s environment which could be affected by different thiol-based oxidative posttranslational modifications (oxiPTMs), since besides *S*-nitrosation, there are other PTMs, such as *S*-sulfenylation, *S*-glutathionylation, and persulfidation, that are mediated by H_2_O_2_, GSH, and H_2_S respectively [92]. They are potential competitors among themselves for a certain thiol group, and the final effect will be a consequence of the relative abundance of these molecules near the target protein. In the case of pepper fruit, a very active nitro-oxidative metabolism has been described during ripening, so this mechanism of S-nitrosation must be considered as a post-translational mechanism for regulating POD IV, which allows it to maintain its activity under the nitro-oxidative cellular stress.

## 4. Conclusions

The present data provide, to our knowledge, the first characterization of the *POD* genes in pepper plants with special emphasis on fruit. In this organ, a total of 10 *POD* genes were identified and were differentially modulated during ripening and by the exogenous application of NO gas. On the other hand, the characterization of POD isozymatic activity allowed to identify four isozymes, with CaPOD II being the most prominent whereas CaPOD IV was the most significantly affected by NO-derived molecules as well as by reducing agents. This suggests that this isozyme should have a key relevance during the ripening of pepper fruits, as well as being a NO target.

## Figures and Tables

**Table 1:** Summary of the 75 peroxidases (*POD*) genes identified in the pepper (*C. annuum* L.) genome, and some of the theoretical molecular properties related to the protein encoded by these genes. They include the number of amino acids (aa), molecular mass of the subunit size (kDa), and their subcellular localization. The ten *CaPOD* genes specifically detected in the sweet pepper fruit transcriptome are highlighted in red. Chr.: chromosome number.

Gene Name	Loc ID	Chr.	Genomic Location	Protein ID	Length (aa)	kDa	Subcellular Localization
*CaPOD1*	107856092	1	32671822–32673357	XP_016556569.1	333	36.7	Plastid
*CaPOD2*	107874082	1	45236930–45240796	XP_016576451.1	326	35.6	Plastid
*CaPOD3*	107874093	1	45249650–45253391	XP_016576460.1	325	35.0	Extracellular/Cytosol
*CaPOD4*	107874527	1	45319089–45322294	XP_016576787.1	330	36.0	Plastid/Vacuole
*CaPOD5*	124898652	1	181026768–181036485	XP_047268295.1	316	35.2	Plastid
*CaPOD6*	107857270	1	213795233–213805050	XP_016557678.1	343	38.5	Extracellular
*CaPOD7*	107850917	1	252561266–252562231	XP_016551241.1	321	35.5	Cytosol/Extracellular
*CaPOD8*	107860974	2	84157374–84161490	XP_047262346.1	325	35.5	Extracellular
*CaPOD9*	107861195	2	126799675–126803192	XP_016562044.1	349	38.5	Plastid
*CaPOD10*	107854094	2	132359051–132361632	XP_016554572.2	365	38.6	Plastid/Vacuole
*CaPOD11*	107859584	2	134101328–134102739	XP_016560122.2	327	37.2	Extracellular
*CaPOD12*	107861283	2	136356851–136358760	XP_016562113.1	330	36.9	Extracellular
*CaPOD13*	107861303	2	138090335–138091718	XP_016562131.1	314	34.1	Extracellular
*CaPOD14*	107859813	2	138097485–138098968	XP_016560411.1	317	34.9	Plastid
*CaPOD15*	107860014	2	139754471–139756109	XP_047263156.1	330	36.1	Plastid
*CaPOD16*	107860013	2	139760596–139762112	XP_016560694.2	332	36.3	Plastid
*CaPOD17*	107860012	2	139766060–139767571	XP_016560693.2	332	36.8	Plastid
*CaPOD18*	107860047	2	141519183–141522229	XP_016560733.1	346	32.3	Vacuole/Extracellular
*CaPOD19*	107860206	2	144557158–144559429	XP_016560965.1	329	36.1	Plastid/Vacuole
*CaPOD20*	107860325	2	146620258–146621615	XP_016561130.1	325	35.7	Extracellular
*CaPOD21*	107860606	2	151534058–151535050	XP_016561495.1	330	36.8	Plastid
*CaPOD22*	107860772	2	153605126–153606707	XP_016561732.2	325	35.4	Extracellular/Vacuole
*CaPOD23*	107860773	2	153608247–153609671	XP_016561733.2	324	35.2	Plastid/Golgi
*CaPOD24*	107863896	3	41679815–41681218	XP_016565574.1	334	36.1	Plastid
*CaPOD25*	107852267	3	166523190–166526792	XP_016552813.1	348	38.2	Plastid
*CaPOD26*	107863356	3	201558537–201559836	XP_016564726.2	317	34.2	Extracellular
*CaPOD27*	107863246	3	217741244–217743566	XP_016564563.2	319	34.4	Plastid
*CaPOD28*	107855938	3	234661502–234663111	XP_016556426.1	321	34.8	Cytosol
*CaPOD29*	107869901	4	1635680–1637002	XP_016571788.2	342	36.9	Extracellular
*CaPOD30*	124897629	4	9153683–9159042	XP_047266625.1	334	36.2	Extracellular
*CaPOD31*	107868171	4	18957575–18962323	XP_047267412.1	334	37.1	Extracellular
*CaPOD32*	107868170	4	18997969–19003730	XP_016570262.1	336	37.3	Extracellular
*CaPOD33*	107867709	4	173753971–173756793	XP_016569561.1	334	37.1	Peroxisome
*CaPOD34*	107867619	4	192580931–192584741	XP_016569421.1	350	38.7	Plastid
*CaPOD35*	107867617	4	192606041–192608813	XP_016569419.1	358	39.5	Extracellular/Vacuole
*CaPOD36*	107853405	4	217269116–217271921	XP_016553885.1	328	36.1	Extracellular
*CaPOD37*	107867212	4	221465603–221468812	XP_016568845.1	341	37.9	Nucleus
*CaPOD38*	107869386	4	229843373–229846291	XP_016571405.1	317	34.8	Vacuole
*CaPOD39*	107853865	5	14775170–14776405	XP_016554339.1	319	34.3	Plastid/Vacuole
*CaPOD40*	107853869	5	14780107–14781396	XP_047268512.1	322	34.5	Plastid
*CaPOD41*	107860938	5	14829467–14830757	XP_016561858.1	322	34.6	Plastid
*CaPOD42*	107866093	5	14861775–14863048	XP_016567762.2	323	35.0	Plastid
*CaPOD43*	107866094	5	14866055–14867328	XP_016567763.1	323	34.9	Plastid
*CaPOD44*	107866095	5	14892113–14893410	XP_016567764.2	322	34.9	Plastid
*CaPOD45*	107866096	5	14942012–14943277	XP_016567765.1	322	34.8	Plastid
*CaPOD46*	107866097	5	14959711–14960996	NP_001311841.1	322	34.7	Plastid
*CaPOD47*	107872526	5	164460717–164465181	XP_016574676.1	315	33.6	Plastid
*CaPOD48*	107871140	5	207103981–207107624	NP_001311926.1	324	34.9	Plastid
*CaPOD49*	107871376	5	219482810–219484153	XP_016573785.2	329	36.1	Plastid
*CaPOD50*	107874883	6	180903059–180904045	XP_016577077.1	328	36.0	Extracellular
*CaPOD51*	107875418	6	224768131–224770315	XP_016577619.1	319	34.3	Extracellular
*CaPOD52*	107877215	7	172378813–172380213	XP_016579397.1	331	35.9	Plastid
*CaPOD53*	107878262	7	193247121–193248773	XP_016580668.1	328	36.0	Plastid
*CaPOD54*	107872642	8	156429677–156430944	XP_016574759.2	332	36.2	Plastid
*CaPOD55*	107840010	8	161207604–161210209	XP_016539187.2	400	34.7	Vacuole
*CaPOD56*	107840027	8	161517334–161518904	NP_001311508.1	332	36.1	Platid
*CaPOD57*	107840318	8	167555454–167557196	XP_016539637.1	329	36.4	Extracellular
*CaPOD58*	107842218	9	9989788–9993702	XP_047252040.1	323	15.7	Plastid
*CaPOD59*	107877245	9	194786339–194789611	XP_016579420.2	328	36.1	Plastid
*CaPOD60*	107877246	9	194812949–194816310	XP_016579421.1	324	35.3	Extracellular
*CaPOD61*	107841908	9	195714065–195715731	XP_016541245.1	322	35.2	Extracellular
*CaPOD62*	107841822	9	218350975–218353270	XP_016541157.1	317	35.0	Plastid
*CaPOD63*	107844910	10	147112017–147118017	XP_047253817.1	319	30.5	Extracellular
*CaPOD64*	107844773	10	150704727–150706012	XP_016544608.2	317	34.7	Extracellular
*CaPOD65*	107844563	10	197101087–197107136	XP_047253737.1	305	34.3	Cytoskeleton
*CaPOD66*	107843551	10	202596247–202600521	XP_016543351.2	329	37.4	Plastid
*CaPOD67*	107844720	10	220335171–220338346	XP_016544563.1	331	36.0	Plastid
*CaPOD68*	107852745	11	6631728–6635289	XP_016553271.2	349	38.7	Extracellular
*CaPOD69*	107847025	11	39881353–39882360	XP_016546761.1	335	37.2	Extracellular
*CaPOD70*	107852613	11	208823755–208825290	XP_016553130.2	326	34.7	Plastid
*CaPOD71*	107851630	12	7151271–7153825	XP_016552197.1	337	37.8	Plastid/Extracellular
*CaPOD72*	107849754	12	221606397–221607065	XP_047256950.1	341	37.5	Plastid
*CaPOD73*	124885353	12	222343207–222343476	XP_047257150.1	319	34.9	Vacuole
*CaPOD74*	107849622	12	227194776–227198629	XP_016549663.1	339	37.1	Plastid
*CaPOD75*	107877264	--	3993–5513	XP_016579433.1	329	35.8	Extracellular

## Data Availability

Sequence Read Archive (SRA) data are available at the following link https://www.ncbi.nlm.nih.gov/sra/PRJNA668052 (accessed on 28 May 2020).
